# Affectionate touch and diurnal oxytocin levels: An ecological momentary assessment study

**DOI:** 10.7554/eLife.81241

**Published:** 2023-05-30

**Authors:** Ekaterina Schneider, Dora Hopf, Corina Aguilar-Raab, Dirk Scheele, Andreas B Neubauer, Uta Sailer, René Hurlemann, Monika Eckstein, Beate Ditzen

**Affiliations:** 1 Institute of Medical Psychology, Center for Psychosocial Medicine, Heidelberg University Hospital Heidelberg Germany; 2 Heidelberg University Heidelberg Germany; 3 Department of Social Neuroscience, Faculty of Psychology, Ruhr University Bochum Bochum Germany; 4 Department for Education and Human Development, DIPF|Leibniz Institute for Research and Information in Education Frankfurt Germany; 5 Center for Research on Individual Development and Adaptive Education of Children at Risk Frankfurt Germany; 6 Department of Behavioural Medicine, Faculty of Medicine, Institute of Basic Medical Sciences, University of Oslo Oslo Norway; 7 Department of Psychiatry, University of Oldenburg Bad ZwischenahnOldenburg Germany; University of Würzburg Germany; University Medical Center Hamburg-Eppendorf Germany

**Keywords:** affectionate touch, oxytocin, cortisol, well-being, Covid-19, Human

## Abstract

**Background:**

Affectionate touch, which is vital for mental and physical health, was restricted during the Covid-19 pandemic. This study investigated the association between momentary affectionate touch and subjective well-being, as well as salivary oxytocin and cortisol in everyday life during the pandemic.

**Methods:**

In the first step, we measured anxiety and depression symptoms, loneliness and attitudes toward social touch in a large cross-sectional online survey (N = 1050). From this sample, N = 247 participants completed ecological momentary assessments over 2 days with six daily assessments by answering smartphone-based questions on affectionate touch and momentary mental state, and providing concomitant saliva samples for cortisol and oxytocin assessment.

**Results:**

Multilevel models showed that on a within-person level, affectionate touch was associated with decreased self-reported anxiety, general burden, stress, and increased oxytocin levels. On a between-person level, affectionate touch was associated with decreased cortisol levels and higher happiness. Moreover, individuals with a positive attitude toward social touch experiencing loneliness reported more mental health problems.

**Conclusions:**

Our results suggest that affectionate touch is linked to higher endogenous oxytocin in times of pandemic and lockdown and might buffer stress on a subjective and hormonal level. These findings might have implications for preventing mental burden during social contact restrictions.

**Funding:**

The study was funded by the German Research Foundation, the German Psychological Society, and German Academic Exchange Service.

## Introduction

Social integration and close social contact have been shown to improve mental and physical health as well as increase longevity (Holt-Lunstad, 2018). This effect has been suggested to be mediated through physical proximity and affectionate touch, with touch serving as a social safety signal (Eckstein et al., 2020). Affectionate touch has been associated with beneficial effects on human development and psychological well-being throughout the lifespan (Atzil et al., 2018; Cascio et al., 2019). Touch activates reward-related brain regions (Kreuder et al., 2017) and reduces stress-induced cortisol (Ditzen et al., 2007) and pain (Kreuder et al., 2019). On a neuroendocrine level, the stress-buffering effects of affectionate touch on subjective measures and activity of the hypothalamic–pituitary–adrenal (HPA) axis have been hypothesized to be mediated by the neuropeptide hormone oxytocin (Eckstein et al., 2020).

The outbreak of the Covid-19 pandemic was a continuous stressor with major health and societal consequences (Fancourt et al., 2021; Mata et al., 2021; Pierce et al., 2020). Immediate restrictions and physical distancing were necessary measures to control the spread of the virus. The resulting physical isolation has been linked to higher self-reported loneliness, especially as a result of the first lockdown (Fancourt et al., 2021; Mata et al., 2021; Pierce et al., 2020) and in individuals with previous higher loneliness (Bu et al., 2020). In general, loneliness and social isolation have been associated with poorer mental and physical health as well as increased mortality (Lee et al., 2021; Leigh-Hunt et al., 2017). Thus, it is not surprising that several recent studies emphasize the potential impact of loneliness during the Covid-19 pandemic on mental health (Brooks et al., 2020; Campion et al., 2020; Rozenkrantz et al., 2020). Large population-based studies suggest that levels of mental distress with clinical significance increased from 18.9% in 2018–19 to 27.3% during the pandemic (Pierce et al., 2020), and the increase was most prominent in the initial phase of the lockdown (Chandola et al., 2022). On the other hand, perceived social support as well as frequent social contact during the pandemic were associated with lower depression scores (Sommerlad et al., 2021).

The request to minimize social contact and increase physical distance during lockdown consequently led to less physical contact and lower frequency of interpersonal touch, potentially increasing the feeling of longing for touch. Moreover, higher longing for touch was associated with prolonged and more severe Covid-19 restrictions (Meijer et al., 2022). Literature on touch deprivation suggests that a lack of touch is associated with lower levels of general well-being and an increased risk of mental health problems (Banerjee et al., 2021). A recent study by von Mohr and colleagues showed that self-reported deprivation of intimate touch (but not other types such as friendly or professional touch) during the Covid-19 lockdown was associated with higher loneliness scores. In addition, they found that intimate touch deprivation was associated with higher anxiety levels; however, this association was no longer significant when accounting for loneliness (von Mohr et al., 2021). The authors suggested that the lack of intimate touch may increase anxiety in individuals with higher loneliness. Burleson and colleagues reported that reduced affectionate touch was associated with more psychological distress, especially for those participants, who typically use touch for affect regulation (Burleson et al., 2022).

Initial laboratory research has demonstrated that receiving touch such as a massage can have beneficial effects evident in reduced self-reported anxiety and stress levels (Kirschner and Kirschner, 2019), as well as decreased cortisol (Maratos et al., 2017) and increased oxytocin (Morhenn et al., 2012) concentrations. Similarly, a more recent study found a significant increase in plasma oxytocin and corresponding neural responses after a foot massage. Interestingly, basal oxytocin concentrations, as well as oxytocin increase after the massage were associated with more positive attitudes toward social touch (Li et al., 2019). Moreover, touching a dog as compared to merely observing it was associated with not only decreased self-reported stress, but also increased self-reported happiness (Sokal et al., 2021). Based on these findings, we hypothesized that a positive attitude toward touch and increased loneliness would be associated with higher anxiety and depression symptoms during the lockdown. On a momentary level, we expected that affectionate touch would be associated with decreased subjective anxiety, distress, and decreased HPA axis activity (cortisol levels), as well as with higher endogenous oxytocin levels. Furthermore, we expected that the link between subjective anxiety and distress with affectionate touch would be mediated by elevated oxytocin levels. To the best of our knowledge, there has not yet been a study investigating the associations of affectionate touch with mental health and neuroendocrine variables during the Covid-19 lockdown. We addressed this gap using ecological momentary assessment (EMA) of both repeated psychological and endocrine measures in a large sample with frequent repeated everyday life measuring.

## Methods

For this study, ethical approval was granted from the ethics committee of the Heidelberg University Medical Faculty (approval no. S-214/2020), and the study was registered online at https://drks.de/search/en/trial/DRKS00021671. All participants provided written informed consent. We used the disclosure of interest form of the International Committee of Medical Journal Editors (ICMJE) to report no conflicts of interest. STROBE protocol was used to standardize reporting.

### Study design and population

In a large online survey launched in April 2020, structural social factors, such as housing situation, anxiety, and depressive symptoms, as well as subjective psychosocial burden, loneliness, and the perception of touch during the physical distancing measures, were assessed (Hopf et al., 2022). Study participants were recruited via local newspapers, radio programs, and social media. In an attempt to more actively involve the study participants in the research (collecting data, carrying out measurements in open formats, reporting unexpected results, i.e. citizen science approach), all participants (N = 1050) who had completed the online survey were invited to take part in a 2-day psychobiological EMA. Participants were given standardized instructions via phone on how to use their smartphones to collect momentary subjective data, as well as saliva samples via a passive drool method at six time points per day over the course of two consecutive days (i.e. in total, each individual provided 12 saliva samples). They received the collecting devices via mail along with the informed consent documents to sign. Sampling times on each day were adapted to the individual wake-up time and were taken directly after awakening, 30 min after, 45 min after, 2½ hr after, 8 hr after, and directly before going to sleep. To reduce potential missing values, minimize irregularities, and increase adherence, the data sampling was monitored by study members.

### Measures

#### Hospital Anxiety and Depression Scale (HADS)

General psychological distress was assessed using the total score of the Hospital Anxiety and Depression Scale (HADS) (Hinz and Brähler, 2011). Sum scores were calculated for anxiety and depression subscales as well as for the total score. The internal consistency of the global HADS score in our data was high (HADS total score: Cronbach’s *α* = 0.89; HADS Anxiety subscale: Cronbach’s *α* = 0.82; HADS Depression subscale: Cronbach’s *α* = 0.82).

### UCLA Loneliness Scale

Loneliness was measured using the 20-item UCLA Loneliness Scale (Döring and Bortz, 1993). Participants rated how often they felt in a certain way during the past 2 wk, with higher scores indicating higher levels of loneliness. The sum scores were used for statistical analyses. In our sample, the scale showed a high internal consistency (Cronbach’s *α* = 0.91).

### Social Touch Questionnaire (STQ)

To measure attitudes toward social touch, we used the Social Touch Questionnaire (STQ) (Wilhelm et al., 2001), assessing different aspects of social touch such as touch involving family and friends vs. touch involving strangers, touch occurring in different settings, as well as touch with sexual vs. without sexual connotation. Internal consistency in our data was high with Cronbach’s *α* = 0.84. Low values of STQ indicate a high liking of social touch, whereas high values indicate a high aversion to social touch. To interpret the results more intuitively, individual scores of the STQ were inverted (i.e. high STQ values indicate a more positive attitude towards touch).

### Ecological momentary assessment

Momentary levels of well-being (anxiety, stress, general and Covid-19 related burden, as well as happiness levels) were assessed through single items (‘Please indicate how you feel at the moment …’) using visual analog scales from 0 (not at all) to 100 (very much). Affectionate touch was assessed with the question ‘Since the last time point, did you experience touch, hugs, kisses, cuddles, etc.?’ and an additional visual analog scale for the intensity rating of the experienced touch from 0 (low intensity) to 100 (high intensity).

### Neuroendocrine measures

On seeing the prompt on their smartphones, participants self-sampled their saliva into Salicaps (small plastic tubes) via passive drool and stored each sample immediately after collection in their home freezers. At the end of data collection, the study team personally visited to collect the samples on dry ice. The saliva samples were stored at –80°C until analyses at the Institute of Medical Psychology’s biochemical lab at Heidelberg University Hospital.

For the analyses of endogenous oxytocin concentrations, saliva samples were thawed and centrifuged at 4°C at 1.500 × *g* for 15 min and subsequently analyzed without extraction (50% of the samples in duplicates) following the protocol of oxytocin enzyme-linked immunosorbent assay from Enzo Life Sciences (ELISA; ENZO Life Sciences, Switzerland). The detection limit was 15 pg/ml, and the variation coefficient for intra- and inter-assay precision was 6.12 and 11.13%, respectively. For cortisol analyses, 20% of the samples were analyzed in duplicates and an ELISA from Demeditec Diagnostics (Demeditec Diagnostics, Germany) was used with a reported detection limit of 0.019 ng/ml. Intra- and inter-assay variations in our sample were 2.95 and 7.51%, respectively.

### Statistical analyses

For data processing, IBM SPSS version 27 was used. Statistical analyses were conducted using R studio (R version 4.1.1) and Mplus (version 8.6). We analyzed the relationship of attitudes toward social touch (STQ) and loneliness (UCLA Loneliness) with anxiety and depression symptoms (HADS total) controlling for age, sex, and presence of mental disorder using multiple regression analyses. No violations of general assumptions for multiple regression (linearity, homoscedasticity, normality, and independence of errors) were detected. The total score of HADS, as well as HADS Anxiety and HADS Depression subscales, were included as dependent variables, whereas STQ, UCLA Loneliness, as well as the interaction variable (STQ × UCLA Loneliness) were entered as independent variables into the model. STQ and UCLA Loneliness scores were centered around their respective means. Missing data were deleted listwise.

To test whether affectionate touch was associated with well-being and neuroendocrine markers in everyday life, we conducted multiple hierarchical linear models. To separate within-person and between-person effects, self-reported touch (yes/no) and the intensity of touch were centered around each person’s mean and the person’s mean was centered on the grand mean. First, we included momentary affectionate touch (yes/no) controlling for age, sex, and day as independent variables to predict individual momentary self-reported anxiety, stress, general and Covid-19 related burden, as well as happiness levels in separate models. Subsequently, we analyzed whether the intensity of experienced touch was associated with these momentary psychological states following the same analytical approach. For models including cortisol and oxytocin measures as dependent variables, we additionally controlled for body mass index (BMI) and several potential confounders: momentary food and drink intake, alcohol, caffeine, and cigarette consumption, as well as physical activity, sleep duration, and quality, problems falling asleep, intake of sleeping pills, forced awakening, and brushing teeth. Furthermore, we controlled for assessment time points by including time (coded from 0 to 3 for the assessment time points 3–6) to control for linear diurnal changes after the awakening response (Ning and Luo, 2017). Additionally, for these models, we conducted random slope models and compared the fit of these models to models without random slope for the focal predictor (touch; touch intensity) using likelihood ratio tests. For the models on affectionate touch as a binary variable (yes/no), we report random intercept and random slopes models in the ‘Results’ section since these showed a statistically better fit compared to random intercept and fixed slopes models. However, for the dimensional intensity of affectionate touch, we report random intercept and fixed slopes models since the random slope models did not yield a better model fit. Before analyses, cortisol and oxytocin levels were log-transformed (natural logarithm) to normalize the distribution. Cortisol and oxytocin awakening response was calculated using the formula for calculating the area under the curve concerning increase (Pruessner et al., 2003).

## Results

### Sample characteristics

**Figure 1.**
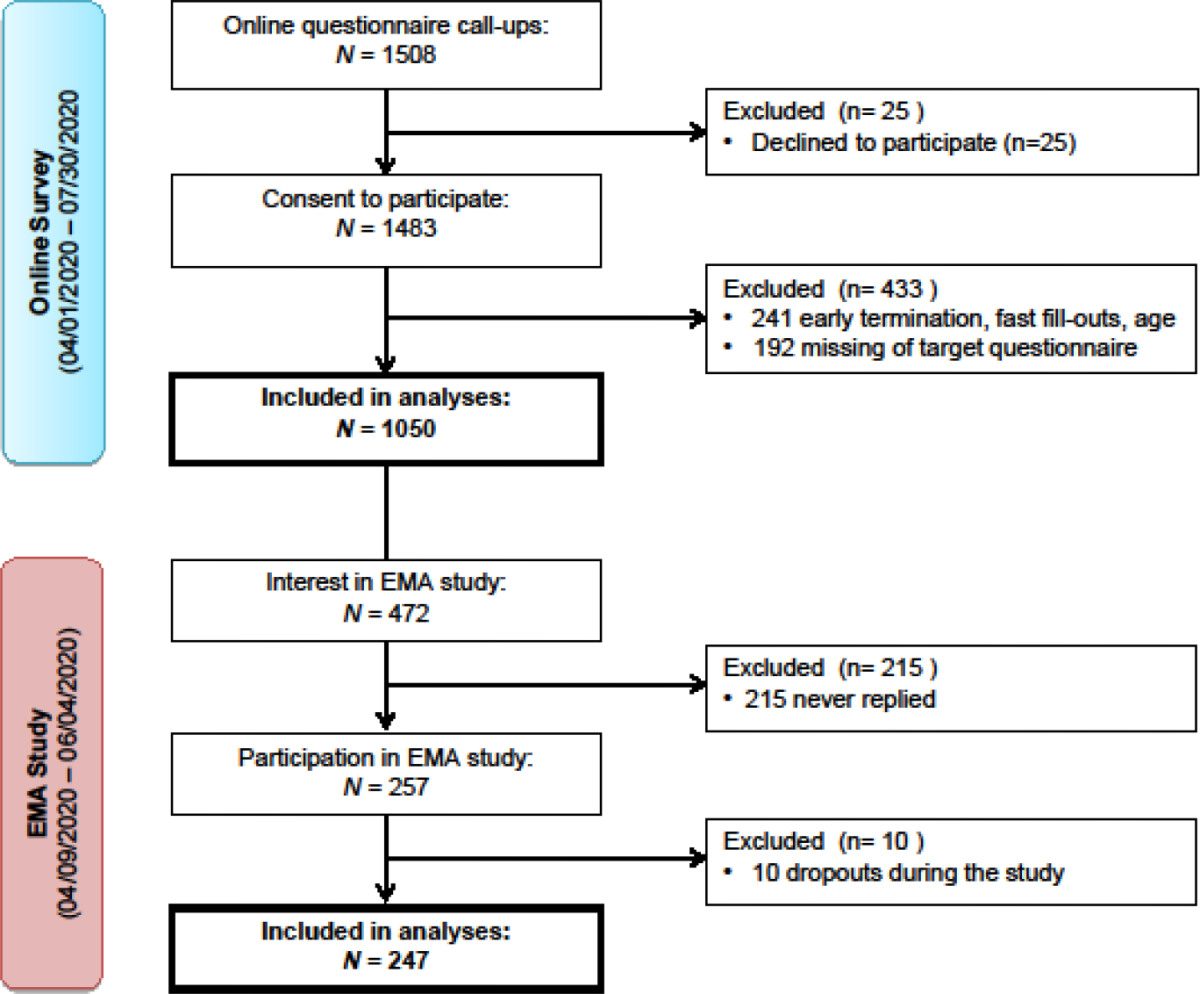
Flowchart of the recruitment process. Figure 1 depicts the recruitment stages of both the online and the ecologically momentary assessments (EMA) study. Participants were recruited between April 1 and July 30, 2020, via online media and local newspapers. Inclusion criteria: fluency in German, minimum age of 18 y, and willingness to participate voluntarily. In total, 1483 individuals agreed to participate, of whom 1050 participants filled out the online questionnaires of interest. Out of the 472 participants who were interested in the EMA study, 247 finished the assessments.

From April to August 2020, 1483 participants filled out the online survey, of whom 433 were excluded from data analysis (see Figure 1). A total of 1050 participants (n = 815 women, n = 227 men, n = 4 non-binary, n = 4 no information on gender) were included in the analyses. Participants’ age ranged from 18 to 81 y, with a mean age of 36.34 (SD = 14.77). 20.2% (n = 212) indicated that they suffered from a diagnosed mental disorder. The most frequent single diagnosis was depression (35%) followed by anxiety disorders (10%). Of those with at least one diagnosis, 27.5% indicated having multiple diagnoses.

After completion of the online survey, 472 individuals indicated that they were interested in the EMA study, of whom 257 confirmed their participation after receiving detailed information. Ten participants withdrew from the study due to personal reasons, resulting in a total of 247 participants (n = 173 women, n = 74 men) completing the 2 d EMA. The mean age of the sample was 32.02 y (SD = 13.12) ranging from 18 to 78 y (for more details on sample characteristics, please see Table 1).

**Table 1.** Sample characteristics of online survey and ecological momentary assessment.

*Sample characteristics of online survey participants*				
	**Men (n = 227**)	**Women (n = 815**)	**Non-binary (n = 4**)	**Missing (n = 4**)
	**M (SD)**	**M (SD)**	**M (SD)**	**M (SD)**
Age (years)	34.67 (15.18)	36.74 (14.58)	45.50 (24.73)	40.25 (15.39)
General psychological distress*	10.22 (6.75)	13.18 (7.49)	21.00 (5.89)	19.50 (14.66)
Anxiety^†^	5.15 (3.68)	6.95 (4.12)	10.50 (3.11)	9.25 (7.68)
Depression ^‡^	5.07 (3.55)	6.22 (4.10)	10.50 (3.32)	10.25 (8.18)
Loneliness ^§^	37.18 (10.15)	39.33 (10.95)	53.00 (13.24)	47.50 (19.50)
Attitude toward social touch ^¶^	33.58 (10.18)	34.76 (12.27)	38.50 (23.39)	40.75 (16.92)
*Sample characteristics of ecological momentary assessment participants*				
	**Men (n = 74**)		**Women (n = 173**)	
	**M**	**SD**	**M**	**SD**
Age (years)	30.99	13.62	33.05	12.41
Cortisol (ng/ml) **	8.40	2.02	8.68	2.31
Oxytocin (pg/ml)**	176.12	106.18	164.54	96.74
Covid-19-related burden**	36.98	24.61	41.78	23.49
General burden	39.96	23.89	47.20	21.78
Stress levels^††^	29.49	15.88	35.62	17.00
Anxiety levels^††^	18.39	15.98	24.14	20.08
Happiness levels^††^	71.13	17.09	67.87	18.42
Intensity of affectionate touch^††^	65.21	20.00	56.57	23.13

Table depicts means (M) and standard deviations (SD). Number of participants indicated as (n).

* Hospital Anxiety and Depression Scale (HADS total score).

† HADS Anxiety subscale.

‡ HADS Depression subscale.

§ University of California, Los Angeles Loneliness Scale (UCLA Loneliness).

¶ Social Touch Questionnaire (STQ).

** Out of 2964 possible data points, n = 2724 remained for analysis after excluding outliers, samples that were not stored as instructed or below detection limit, sampling problems.

†† Momentary self-reported state.

### Attitude toward touch and its association with anxiety, depression, and loneliness

On average, participants’ HADS total scores were M = 12.58 (SD = 7.49, range = 0–37). 39.7% of the sample had values above the cut-off score (>13) compared to a reference sample (Hinz and Brähler, 2011). The average HADS Anxiety subscale score was M = 6.58 (SD = 4.19, range = 0–20), whereas the HADS Depression subscale score was M = 5.99 (SD = 4.04, range = 0–21) with values exceeding the cut-off scores (>8) in 29.8 and 24.5% of cases, respectively. The results of multiple regression analyses showed significant main effects of sex (*β* = 0.111; t(1031) = 4.655, p<0.001), presence of diagnosed mental disorder (β = 0.151; t(1031) = 5.882, p<0.001), UCLA Loneliness (β = 0.548; t(1031) = 20.403, p<0.001), STQ (*β* = -0.052; t(1031) = −2.083, p=0.038), as well as a significant interaction of UCLA Loneliness × STQ (β = 0.052; t(1031) = 2.104, p=0.036) on the outcome variable total HADS score. Thus, anxiety and depression symptoms were higher in women, individuals with a mental disorder and participants with higher loneliness; and lower in participants with a more positive attitude toward touch. In contrast, although the moderation effects were small, they indicate that the association of loneliness with anxiety and depression symptoms was more pronounced for individuals with a more positive attitude toward social touch. The model tested here was significant overall (F(6,1031) = 125.1, p<0.001) with an R² of 0.421.

Next, we analyzed the association of the UCLA Loneliness × STQ interaction with the subscales of the HADS by following the same analytical approach. Here, we found that the outcome variable HADS Anxiety was also significantly and positively associated with female sex (β = 0.142; t(1031) = 5.369, p<0.001), presence of mental disorder (β = 0.180; t(1031) = 6.3, p<0.001), and UCLA Loneliness (β = 0.404; t(1031) = 13.54, p<0.001). Furthermore, we observed a significant interaction of UCLA Loneliness × STQ (β = 0.079; t(1031) = 2.907, p=0.004). However, the subscale HADS Depression showed only a significant association with sex (β = 0.060; t(1031) = 2.593, p=0.010), presence of mental disorder (β = 0.097; t(1031) = 3.838, p<0.001), and UCLA Loneliness (β = 0.603; t(1031) = 22.974, p<0.001). The UCLA Loneliness × STQ interaction was not significant (p=0.539). Both models with the outcome variable HADS Anxiety as well as with HADS Depression were overall significant (F(6,1031) = 69.25, p<0.001; with an R² of 0.287 and F(6,1031) = 139.1, p<0.001; with an R² of 0.447, respectively).

### Affectionate touch, anxiety, oxytocin, and stress-related outcomes on a momentary level

**Figure 2.**
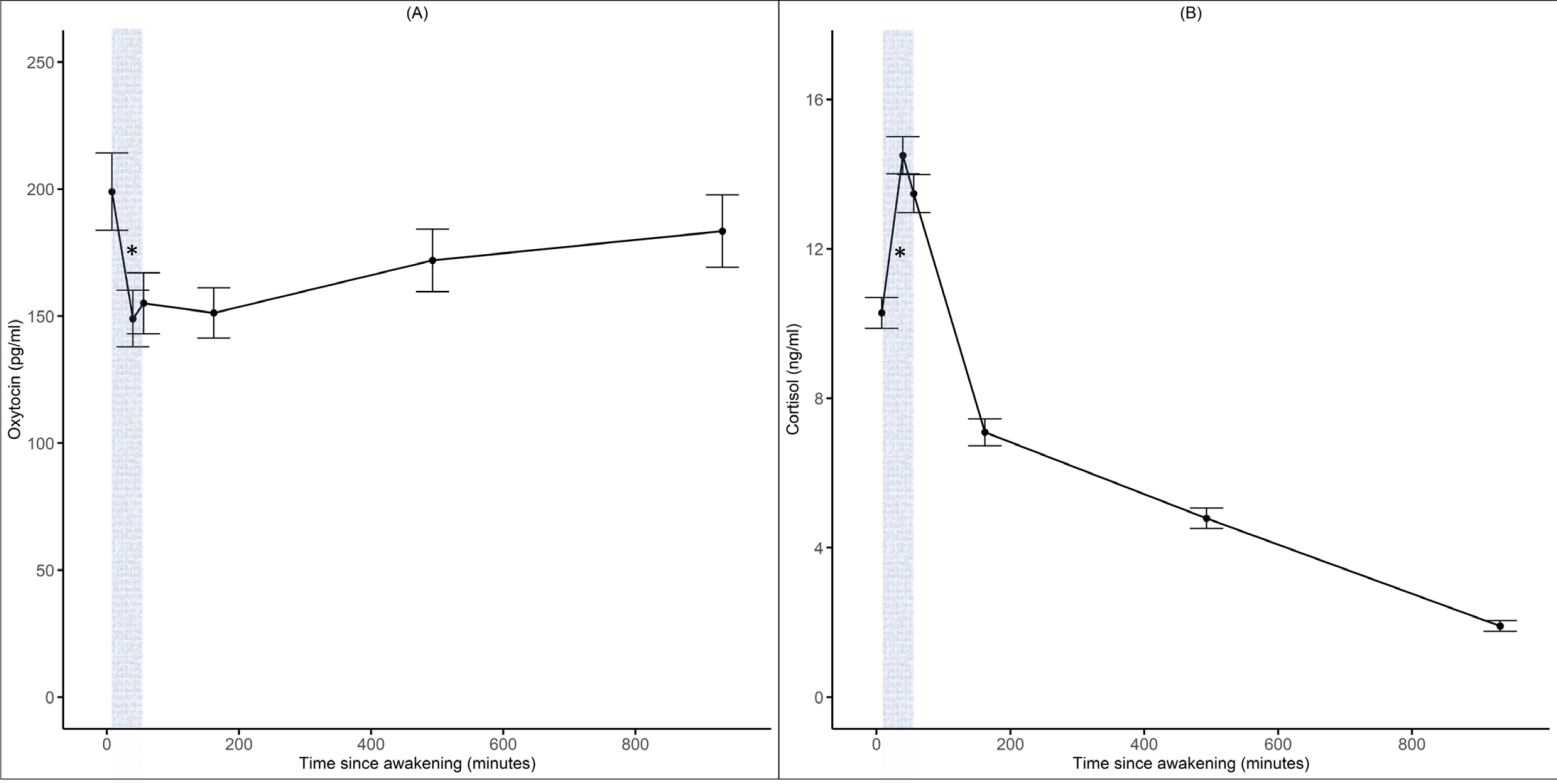
Diurnal oxytocin and cortisol trajectories. Panels (**A**) and (**B**) illustrate the daily oxytocin (pg/ml) and cortisol (ng/ml) trajectories across 2 d and all participants. Gray area indicates cortisol and oxytocin awakening response. Error bars indicate 95% confidence intervals.

Descriptive statistics of outcomes of interest are displayed in Table 1. In addition, an explorative graphical illustration of daily profiles of oxytocin and cortisol shows their variation throughout the day (Figure 2). The patterns of daily profiles did not appear to differ based on participants’ relationship status (single vs. in a relationship) or living situation (alone vs. with others) (see Figure 2—figure supplement 1). A positive correlation between the two assessment days was found for individual (ln-transformed) mean values of oxytocin (r(227) = 0.850, p<0.001), as well as cortisol (r(243) = 0.571, p<0.001) levels. Additionally, we found a significant negative correlation between mean cortisol and oxytocin awakening response (r(181) = –0.195, p=0.008).

**Figure 2—figure supplement 1.**
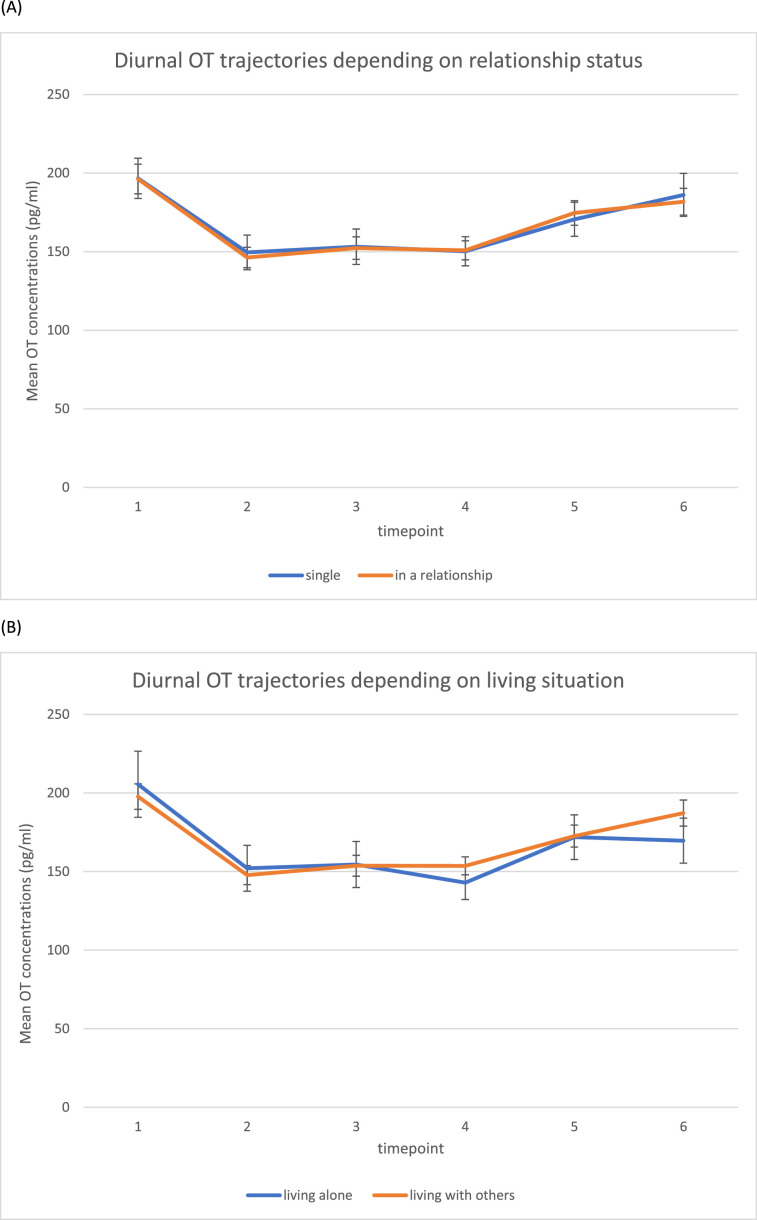
Diurnal oxytocin levels depending on relationship status (**A**) and living arrangements (**B**).

Results from separate random intercept and random slopes multilevel analyses showed that on a momentary (within-person) level, presence of affectionate touch was significantly and negatively associated with stress (b = −4.187; t(793) = −2.100; p=0.036), but not with general burden, anxiety, happiness, cortisol, or with oxytocin levels (see Tables 2 and 3, respectively). The negative association with Covid-19-related burden did not reach statistical significance (b = −2.660; t(792) = −1.867; p=0.062).

**Table 2.** Results of the associations between affectionate touch and self-reported psychological affective states.

‍(A) Random intercept and random slopes models					
Effects	General burden	Covid-19 burden	Stress	Anxiety	Happiness
Fixed effectsWithin-person					
Intercept	47.734 (4.668); p<0.001	43.145 (5.009); p<0.001	34.431 (3.596); p<0.001	21.096 (3.958); p<0.001	71.080 (3.778); p<0.001
‍Touch*	0.462 (1.561); p=0.767	–2.660 (1.424); p=0.062	–4.187 (1.994); p=0.036	–0.217 (1.510); p=0.886	1.557 (1.599); p=0.331
**‍Between-person**					
Touch*	–5.560 (3.791); p=0.144	–7.478 (4.068); p=0.067	–7.534 (2.907); p=0.010	–1.483 (3.210); p=0.645	12.420 (3.068); p<0.001
‍**Covariates**					
‍Age	–0.186 (0.122); p=0.128	–0.158 (0.131); p=0.228	–0.136 (0.094); p=0.150	–0.090 (0.103); p=0.388	0.051 (0.099); p=0.605
‍Sex^†^	4.709 (3.325); p=0.158	3.761 (3.565); p=0.293	4.986 (2.524); p=0.050	6.568 (2.814); p=0.021	–3.318 (2.681); p=0.217
Day	–2.196 (0.864); p=0.011	–2.602 (0.981); p=0.008	–4.189 (1.228); p<0.001	–3.145 (0.875); p<0.001	1.637 (0.901); p=0.070
**‍Random effects (SD)**					
‍Intercept	21.487	22.794	13.694	17.647	16.597
‍Touch*	9.141	0.642	8.094	7.906	8.945
Residual	12.821	14.864	18.741	13.076	13.478
**(B) Random intercept and fixed slopes models**					
**Effects**	**General burden**	**Covid-19 burden**	**Stress**	**Anxiety**	**Happiness**
**Fixed effects** **Within-person**					
Intercept	44.439 (6.130); p<0.001	39.748 (6.114); p<0.001	32.966 (4.609); p<0.001	24.466 (5.277); p<0.001	71.437 (4.626); p<0.001
‍Touch intensity	–0.077 (0.028); p=0.008	–0.068 (0.036); p=0.058	–0.148 (0.044); p<0.001	–0.065 (0.029); p=0.026	0.085 (0.030); p=0.005
**‍Between-person**					
‍‍Touch intensity	–0.121 (0.086); p=0.163	–0.138 (0.087); p=0.115	–0.223 (0.067); p=0.001	–0.102 (0.074); p=0.171	0.314 (0.066); p<0.001
‍**Covariates**					
Age	–0.031 (0.161); p=0.847	–0.082 (0.161); p=0.610	–0.090 (0.125); p=0.475	–0.158 (0.143); p=0.270	0.007 (0.122); p=0.952
‍Sex^†^	2.647 (4.253); p=0.535	4.143 (4.214); p=0.327	2.650 (3.069); p=389	5.932 (3.645); p=0.106	–0.030 (3.171); p=0.993
‍‍Day	–3.788 (1.056); p<0.001	–4.930 (1.302); p<0.001	–4.791 (1.583); p=0.003	–3.718 (1.078); p<0.001	3.695 (1.112); p=0.001
‍**Random effects (SD)**					
Intercept	22.467	21.601	13.206	18.622	15.842
‍Residual	11.812	14.626	18.300	12.121	12.591

Table depicts coefficients (standard errors in parentheses) and p-values of associations between (A) the presence and (B) intensity of affectionate touch and psychological variables. Number of observations = 593–1023, Number of participants 162–227.

* 0 = no, 1 = yes.

† 0 = male, 1 = female.

**Table 3.** Results of the associations between affectionate touch and hormonal levels.

(A) Random intercept and random slopes models			(B) Random intercept and fixed slopes models		
Effects	Cortisol	Oxytocin	Effects	Cortisol	Oxytocin
Fixed effectsWithin-person			Fixed effectsWithin-person		
Intercept	2.941 (0.165); p<0.001	4.973 (0.405); p<0.001	Intercept	2.744 (0.370); p<0.001	4.657 (0.798); p<0.001
‍Touch*	–0.019 (0.060); p=0.756	–0.030 (0.076); p=0.688	Touch intensity	–0.001 (0.001); p=0.367	0.006 (0.002); p=0.003
**‍Between-person**			**Between-person**		
Touch*	–0.121 (0.057); p=0.036	–0.145 (0.147); p=0.329	Touch intensity	–0.001 (0.002); p=0.504	0.002 (0.003); p=0.489
**‍Covariates**			**Covariates**		
‍Age	–0.001 (0.002); p=0.633	–0.013 (0.005); p=0.011	Age	–0.001 (0.003); p=0.776	–0.019 (0.007); p=0.007
‍Sex^†^	–0.020 (0.044); p=0.647	–0.129 (0.123); p=0.293	Sex^†^	–0.001 (0.063); p=0.989	–0.167 (0.151); p=0.272
‍Day	–0.050 (0.035); p=0.155	–0.010 (0.059); p=0.863	Day	–0.032 (0.051); p=0.528	0.077 (0.086); p=0.370
‍Time-fall ^‡^	–0.476 (0.023); p<0.001	0.051 (0.035); p=0.145	Time-fall ^‡^	–0.471 (0.032); p<0.001	0.025 (0.049); p=0.611
‍Body mass index	–0.014 (0.005); p=0.009	0.012 (0.015); p=0.422	Body mass index	0.005 (0.009); p=0.585	0.014 (0.022); p=0.528
‍Eating*	–0.077 (0.075); p=0.302	0.032 (0.117); p=0.788	Eating*	–0.125 (0.099); p=0.210	0.099 (0.149); p=0.506
‍Drinking*	–0.007 (0.081); p=0.933	0.045 (0.126); p=0.722	‍Drinking*	–0.011 (0.108); p=0.919	–0.039 (0.161); p=0.810
‍Caffeine*	0.127 (0.043); p=0.004	–0.099 (0.070); p=0.158	‍Caffeine*	0.131 (0.064); p=0.043	0.157 (0.102); p=0.125
‍Alcohol*	–0.030 (0.063); p=0.628	–0.174 (0.096); p=0.070	‍Alcohol*	–0.012 (0.072); p=0.865	–0.154 (0.112); p=0.171
‍Cigarettes*	0.104 (0.072); p=0.153	0.053 (0.139); p=0.705	Cigarettes*	0.089 (0.098); p=0.368	–0.054 (0.179); p=0.766
‍Physical activity*	0.039 (0.040); p=0.332	0.108 (0.065); p=0.098	‍Physical activity*	–0.072 (0.056); p=0.199	0.014 (0.086); p=0.873
‍Sleep duration ^§^	–0.003 (0.004); p=0.371	–0.009 (0.008); p=0.272	‍Sleep duration ^§^	–0.029 (0.029); p=0.321	0.046 (0.060); p=0.438
‍Sleep quality ^¶^	–0.001 (0.001); p=0.289	–0.000 (0.002); p=0.912	‍Sleep quality ^¶^	–0.001 (0.001); p=0.477	–0.000 (0.002); p=0.960
Problem falling asleep*	0.031 (0.053); p=0.552	–0.245 (0.101); p=0.015	Problem falling asleep*	0.030 (0.081); p=0.709	–0.133 (0.148); p=0.370
Sleeping pills*	0.032 (0.109); p=0.772	0.031 (0.254); p=0.904	Sleeping pills*	–0.203 (0.290); p=0.487	0.771 (0.453); p=0.093
Forced awake*	–0.008 (0.041); p=0.838	0.012 (0.088); p=0.894	Forced awake*	0.016 (0.058); p=0.788	0.075 (0.117); p=0.520
Brushing teeth*	0.045 (0.036); p=0.217	0.096 (0.056); p=0.090	Brushing teeth*	0.029 (0.051); p=0.568	0.016 (0.077); p=0.839
**Random effects (SD)**			**Random effects (SD)**		
‍Intercept	0.149	0.542	Intercept	0.179	0.546
‍Touch*	0.273	0.166	Touch intensity	–	–
‍Residual	0.334	0.477	Residual	0.339	0.461

Table depicts unstandardized coefficients (standard errors in parentheses) and p-values of hormonal associations with (A) the presence and (B) intensity of affectionate touch. Number of observations = 251–545. Number of participants = 88–152.

* 0 = no, 1 = yes.

† 0 = male, 1 = female.

‡ 0 = time point 1–3, 1 = time point 4, 2 = time point 5, 3 = time point 6.

§ In hours.

¶ 1 = very bad, 101 = very good.

**Figure 3.**
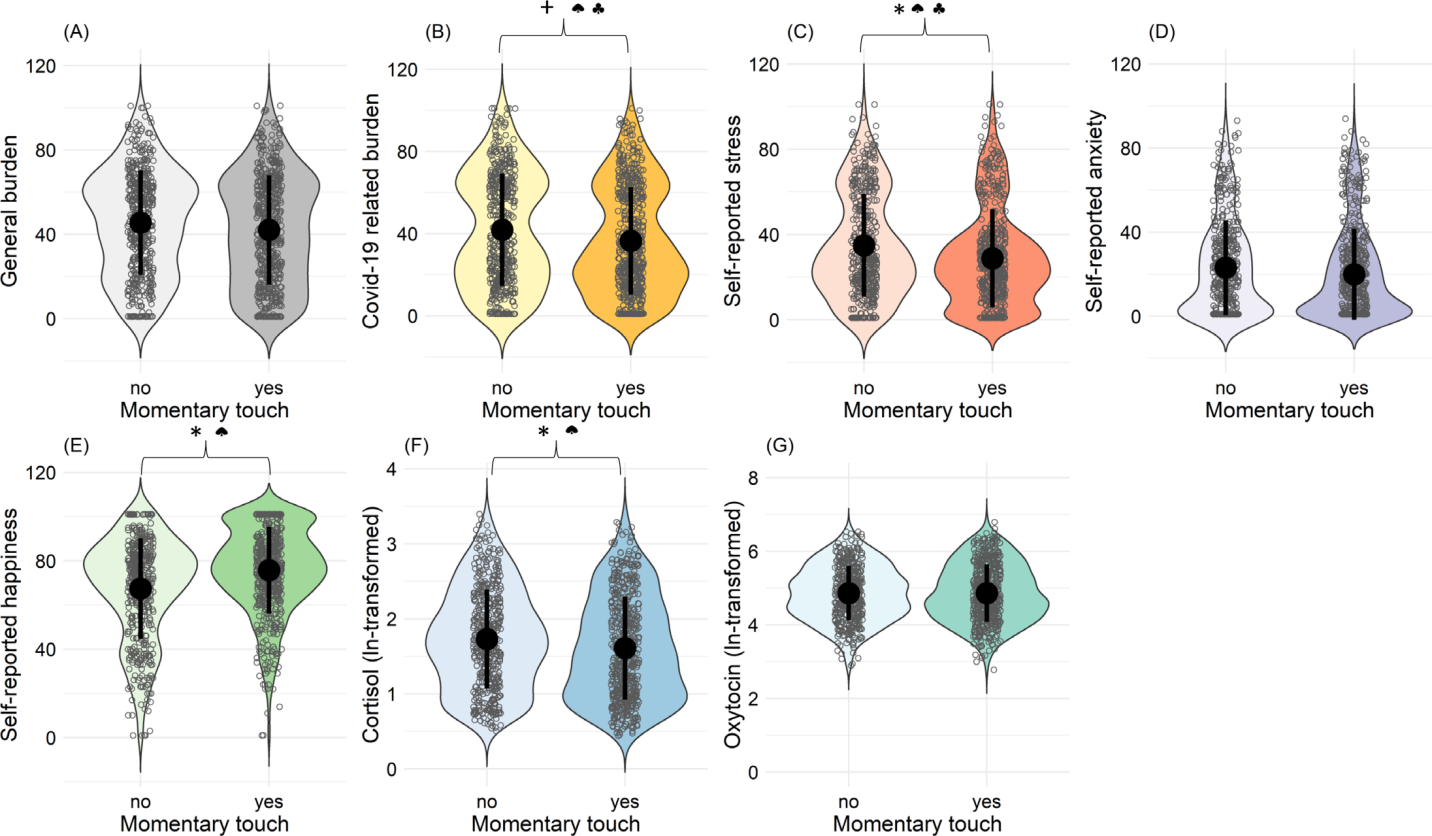
Associations between occurrence of affectionate touch and psychological and hormonal state. Panels (**A**) to (**G**) illustrate violin plots with density distributions of subjective ratings of general and Covid-19-related burden, stress, anxiety, happiness, cortisol, and oxytocin, depending on whether touch occurred or not. Each dot represents one assessment. Central dots (black) represent each mean. Black lines represent the standard deviations. * indicates statistically significant results (p<0.05). +indicates a statistical trend (p<0.1). ♠ indicates statistically significant between-person effect. ♣ indicates statistically significant within-person effect.

On a between-person level, affectionate touch was significantly associated with lower cortisol (b = −0.121; t(128) = −2.118; p=0.036) (see Table 3), stress (b = −7.534; t(223) = −2.592; p=0.010), as well as with higher happiness (b = 12.420; t(223) = 4.049; p<0.001) levels (see Table 2), but not with general burden or anxiety. The negative association with Covid-19-related burden did not reach statistical significance (b = −7.478; t(223) = −1.838; p=0.067) (see Figure 3).

We also analyzed the intensity of experienced affectionate touch as a predictor for psychological and hormonal outcomes, separating within-person and between-person effects. We found that within a person there were significant and negative associations of the intensity of momentary affectionate touch with anxiety (b = −0.065; t(430) = −2.232; p=0.026), stress (b = −0.148; t(430) = −3.363; p<0.001), general burden (b = −0.077; t(430) = –2.687; p=0.008), and positive associations with momentary happiness (b = 0.085; t(430) = 2.795; p=0.005). The negative association with Covid-19-related burden, however, did not reach statistical significance (b = −0.068; t(428) = −1.900; p=0.058) (see Table 2).

Momentary oxytocin levels were significantly higher with more intensive affectionate touch (b = 0.006; t(149) = 3.058; p=0.002) and cortisol levels were descriptively slightly lower; however, this effect was not statistically significant (see Table 3).

**Figure 4.**
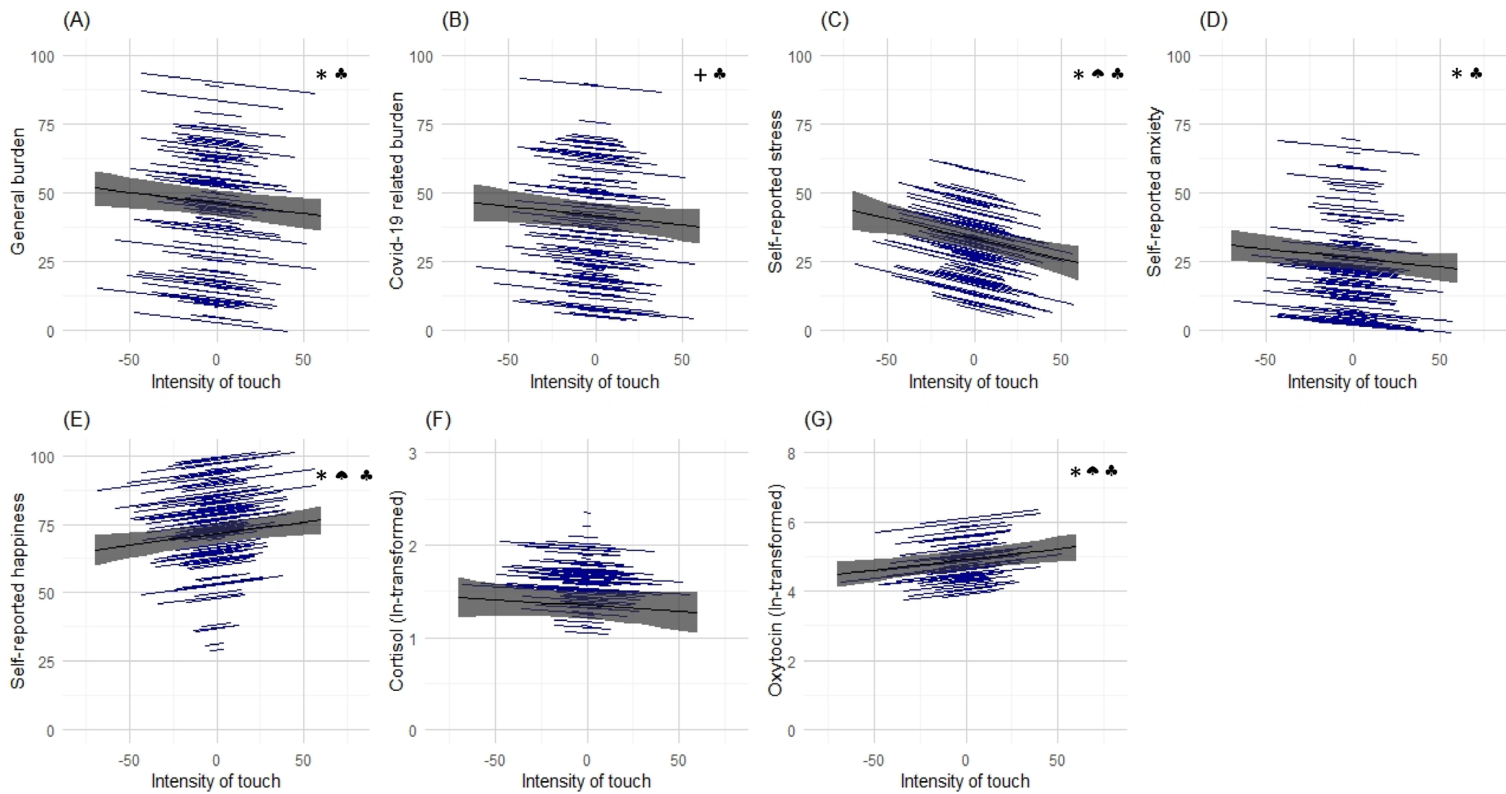
Associations between momentary affectionate touch intensity and psychological and hormonal state. Panels (**A**) to (**G**) illustrate the results of random intercept and fixed slopes models depicting associations of momentary intensity of touch with self-reported general burden and Covid-19 related burden, stress, anxiety, happiness, cortisol, and oxytocin. Gray lines indicate the overall predicted slope, whereas the blue lines indicate the individual’s predicted slopes with their minimum and maximum predicted values as endpoints. Gray areas depict the 95% confidence band. * indicates statistically significant results (p<0.05).+indicates a statistical trend (p<0.1). ♠ indicates statistically significant between-person effect. ♣ indicates statistically significant within-person effect.

Furthermore, on the between-person level, higher intensity of affectionate touch was significantly associated with less stress (b = −0.223; t(158) = −3.318; p=0.001) and greater happiness (b = 0.314; t(159) = 4.764; p<0.001) (see Figure 4), but not with anxiety, general burden, Covid-19-related burden or hormonal levels. No statistically significant sex effects emerged in any of these analyses.

In a final set of analyses, we conducted multilevel structural equation models to investigate whether there was evidence for oxytocin mediating the effects of affectionate touch on cortisol and/or self-report outcomes. None of the indirect effects of the presence of affectionate touch (p>0.773) or intensity of affectionate touch (p>0.194) on the within-person level were statistically significant. Furthermore, on the within-person level, the correlations of momentary oxytocin with cortisol (*r* = −0.016, p=0.521), Covid-19-related burden (*r* = −0.030, p=0.210), stress (*r* = −0.020, p=0.442), anxiety (*r* = −0.013, p=0.660), and happiness (*r* = 0.031, p=0.328) were not statistically significant.

## Discussion

This study investigated the associations of affectionate touch with self-reported mental health and mood, as well as with momentary endogenous oxytocin and cortisol levels during the first Covid-19 lockdown in the spring of 2020.

In our online survey data, we found significant main effects of sex, psychopathology, and loneliness on psychological distress (HADS total score) and, more specifically, on anxiety (HADS Anxiety) and depressive (HADS Depression) symptoms. Individuals reported higher levels of depression and anxiety, especially if they were female or burdened by mental illness or loneliness. These data are in line with previous studies (Fancourt et al., 2021; Mata et al., 2021; Pierce et al., 2020). Interestingly, analyses showed that the attitude toward touch significantly moderated the association between loneliness and the HADS total score, as well as the HADS Anxiety subscale. Thus, individuals with a positive attitude and affect toward social touch experiencing loneliness showed higher distress and anxiety in times of Covid-19-related lockdown. Whether these moderation effects are apparent outside of pandemic-caused physical restrictions is unknown and should be addressed in future studies. These findings support our hypothesis that touch deprivation and loneliness could be related to anxiety symptoms. However, it is further important to note that about 20% of the participants reported having at least one psychiatric diagnosis. In comparison, the pre-pandemic 12 mo prevalence in the general population in Germany is about 28% (Jacobi et al., 2014). Thus, our sample seems to be slightly less burdened compared to the general population, which partly limits the generalizability of the results.

Results of the psychobiological EMA study in a large sample of a broad age range showed that the presence of affectionate touch was negatively associated with stress and cortisol levels and positively linked with happiness. Moreover, the more intensely affectionate touch was experienced, the lower were subsequent subjective anxiety, general burden and stress levels. Higher intensity of affectionate touch was associated with elevated oxytocin and self-reported happiness. Note that in this data assessment in everyday life affectionate touch could not be experimentally manipulated and these correlative results should be interpreted with caution. However, the results could be interpreted that affectionate touch during the Covid-19 pandemic buffers anxiety and stress and downregulates the HPA response, particularly cortisol. At the same time, the intensity of affectionate touch was associated with increased endogenous oxytocin levels and subjective happiness. Interestingly, a recent study demonstrated that foot massage was rated as more pleasurable and rewarding and was associated with a higher increase of oxytocin after the massage administered by hand as compared to machine-administered massage, although the intensity of the massage was rated similarly (Li et al., 2019). Furthermore, the oxytocin system and its potential stress-ameliorating effects seem to be triggered by meaningful and intense touch in particular (Eckstein et al., 2020).

To our knowledge, the present data provide the first empirical evidence to suggest that affectionate touch is related to reduced anxiety, stress, and general burden, as well as stress-responsive cortisol levels and at the same time is linked to higher endogenous oxytocin levels and well-being in an ecologically valid everyday life setting.

Up to now, no systematic data on daily oxytocin profiles and momentary oxytocin levels in a large sample of men and women from varying age groups have been available. Based on single peripheral oxytocin measures from relatively small samples so far (see Valstad et al., 2017, for an overview), it was the object of debate whether peripheral oxytocin levels might be interpretive of emotional functioning or correspond with stressful experiences (Engel et al., 2019). Moreover, in the last decade, there has been an extensive discussion about the reliability and validity of peripheral oxytocin measures (Martins et al., 2020; Szeto et al., 2011; Tabak et al., 2023). There are several methodological issues and challenges associated with measuring oxytocin in blood plasma and saliva samples (Tabak et al., 2023). For example, studies have shown that oxytocin concentrations after sample extraction are much lower compared to unextracted oxytocin measurements (Szeto et al., 2011). Additionally, the correlations between extracted and unextracted oxytocin levels as well as between saliva and plasma oxytocin concentrations have been inconsistent across studies (e.g. Hoffman et al., 2012; Martins et al., 2020; Nagahashi-Araki et al., 2022; Szeto et al., 2011). These inconsistencies might be due to numerous reasons including differences in the study populations, methods of sample processing, and analyses. In particular, using different assay types (e.g. radioimmunoassay vs. enzyme immunoassay), as well as sample preparation (extraction vs. non-extraction), may contribute to these inconsistencies and make it difficult to compare results between studies (Tabak et al., 2023). Since unextracted samples were used in this study, the concentrations probably represent both free and bound oxytocin (MacLean et al., 2019), thereby potentially limiting the comparability with studies using extracted samples.

Another important issue is the intraindividual stability of oxytocin over time (Feldman et al., 2013; Martins et al., 2020; Schneiderman et al., 2012). A recent study reports no correlation of single oxytocin measures between several assessments, indicating that single measures of oxytocin might not be reliable to represent oxytocin baseline levels (Martins et al., 2020). In our sample, we found a significant positive correlation of mean oxytocin values between the two assessment days. As the fluctuations of oxytocin throughout the day were apparent in our study, correlating mean values of six oxytocin measures over the day might represent the individual baseline oxytocin levels better than single measures (this issue has been also discussed in Tabak et al., 2023). However, the difference between our data and the findings reported by Martins et al., 2020 might also reflect methodological differences as previous studies with unextracted samples also reported positive correlations over time (Feldman et al., 2013; Schneiderman et al., 2012).

In our study, we found a positive association between momentary oxytocin levels and the intensity of affectionate touch on a within-person level. Our findings are in line with previous studies showing an increase in salivary oxytocin after self-touch (de Jong et al., 2015), standardized touch (Portnova et al., 2020), and massage (Li et al., 2019; Moussa et al., 2021). Notably, in recent animal research, it has been shown that the density of oxytocin neurons in the brains of male mice increased after social isolation and that oxytocin neurons are involved in regulating social craving (Musardo et al., 2022). Moreover, social touch has been associated with central nervous system oxytocin activation and secretion into the periphery (Tang et al., 2020). Our data are in line with this research; however, we did not find support for the hypothesis that peripheral oxytocin directly mediated the effects of affective touch on momentary subjective distress and well-being on a statistical level. Central nervous oxytocin dynamics (receptor sensitivity, real-time levels, local gene expression, or methylation) and their interaction with the HPA axis cannot be measured in the living human brain presently (Quintana et al., 2019). Thus, although speculative, our results might suggest that central nervous system oxytocin mechanisms as triggered by touch can modulate endocrine outcomes (peripheral oxytocin, cortisol) and subjective distress, as well as well-being. Furthermore, affectionate touch might influence different outcome levels in parallel (not mediated) processes, or the effects of oxytocin on perceived distress, well-being, and cortisol could unfold across a longer time span, instead of on a moment-to-moment basis. Alternatively, it is also possible that initially higher levels of participants’ well-being (including lower stress, anxiety, or burden) might have increased feelings of closeness and therefore promoted affectionate touch. Also, other aspects that accompany physical contact such as eye contact, compliments, or affective closeness with loved ones could have contributed to the beneficial effects.

The study has some limitations that need to be addressed. The assessment of individual depression and anxiety levels was based on the self-reports using the HADS. Although this instrument has been validated and repeatedly used in clinical practice (Hinz and Brähler, 2011), it does not replace clinical interviews and might be influenced by self-report bias. Furthermore, in the EMA measures we used single items to minimize the drop-out rate during the study, but these items might not comprehensively reflect the individual’s experience of burden, stress, anxiety, or happiness. In addition, touch from strangers was restricted during the pandemic; thus, affectionate touch experiences were probably mostly from family contacts. Therefore, we cannot draw differential conclusions about varying contexts of touch. While in general, being touched by strangers may be rated as less pleasant, during times of a pandemic it is also associated with a higher risk for infection. In contrast, during the lockdown, touch at home may be experienced either as harmless and pleasant (Sorokowska et al., 2021) or as too close to feel comfortable during times of limited distraction and constant and close physical contact with family members. The latter is particularly relevant when it comes to the association of touch (yes/no) with cortisol and oxytocin. While touch, per se, seemed to be associated with reduced cortisol levels in the present sample, oxytocin secretion appears to be related to the intensity of touch. However, since this is a cross-sectional study, we here interpret associations rather than causal effects. The Covid-19-related lockdown provided us with a social situation to study the effects of touch between family/household members (romantic couples, parent–child dyads, etc.) in a relatively controlled setting. While the situation was quite specific and limited the generalizability of the results to everyday life in pre- or post-pandemic conditions, the risk of viral infection was not the only concern among the population. Participants reported significant concerns about being isolated from others and how long it might take for them to get back to normal (Hopf et al., 2021). These concerns related, at least in part, to the fear of loneliness, defined as a perceived lack of social connection and the distress this causes (Bekhet et al., 2008). Thus, these results obtained in the general population during pandemic-related restrictions can be partially generalized to other situations, such as a lack of social contacts due to migration, physical illnesses/disabilities, or other reasons.

### Conclusion

Taken together, the present findings provide support for the links of affectionate touch with more positive mental health outcomes during times of prolonged stress. Notably, the above associations with lower anxiety, better mood, and reduced cortisol levels in everyday life during the Covid-19 lockdown showed that more intense affectionate touch is related to higher salivary oxytocin levels on a moment-to-moment basis. This suggests that endogenous oxytocin might be stimulated through targeted behavior (e.g. social touch), which could have implications for prevention and interventions for individuals who are particularly vulnerable during times of stress and social isolation.

## Data Availability

The datasets analyzed and presented in this manuscript are openly available online (https://doi.org/10.11588/data/WFNWJT). The following dataset was generated: SchneiderE HopfD DitzenB 2023Affectionate touch and diurnal oxytocin levels: An ecological momentary assessment study [Research Data]heiDATA10.11588/data/WFNWJTPMC1022911237252874

## Additional files

MDAR checklist
