# Supplementary material for: Affectionate touch and diurnal oxytocin levels: An ecological momentary assessment study
Source: eLife. 2023 May 30;12:e81241. doi: 10.7554/eLife.81241 (PMC10229112; doi:10.7554/eLife.81241)
Supplement: Peer review history

# Editor report: Editor's evaluation

**DOI:** 10.7554/eLife.81241.sa0

**Authors:** Matthias Gamer

**Affiliations:** https://ror.org/00fbnyb24 University of Würzburg Germany

This important study combines a large cross-sectional survey with detailed ecological momentary assessment to examine the relationship between affectionate touch and well-being during the first wave of the COVID-19 pandemic in Germany. The manuscript reports valuable and solid findings extending previous research in this domain. Specifically, the combination of ecologically momentary assessment data with repeated measurements of salivary cortisol and oxytocin adds to the current understanding of how affectionate touch relates to psychological burden and affect. Due to its correlational nature, the causality of effects remains speculative and needs to be addressed by future work.

# Decision letter

**DOI:** 10.7554/eLife.81241.sa1

**Affiliations:** https://ror.org/00fbnyb24 University of Würzburg Germany; https://ror.org/00fbnyb24 University of Würzburg Germany; https://ror.org/04qr3zq92 University of Electronic Science and Technology of China China

In the interests of transparency, eLife publishes the most substantive revision requests and the accompanying author responses.

**Decision letter after peer review:**

Thank you for submitting your article "Affectionate touch and diurnal oxytocin levels: An ecological momentary assessment study" for consideration by *eLife*. Your article has been reviewed by 3 peer reviewers, and the evaluation has been overseen by a Reviewing Editor and Christian Büchel as the Senior Editor. The following individuals involved in review of your submission have agreed to reveal their identity: Matthias Gamer (Reviewer #1); Elizabeth Anne Broadbent (Reviewer #2); Keith M Kendrick (Reviewer #3).

The reviewers have discussed their reviews with one another, and the Reviewing Editor has drafted this to help you prepare a revised submission.

Essential revisions:

1) The interpretation and conclusions of this study should be toned down as this purely correlative approach does not allow for drawing causal inferences about the relationship between affectionate touch, anxiety, stress, and hormone levels.

2) It should be acknowledged that different types of affectionate touch and contexts might differentially affect wellbeing and hormone levels. Furthermore, other social and physical factors might have been affected by the restrictions during the first wave of the COVID-19 pandemic in Germany. Such potential confounds should be discussed in more detail with respect to the generalizability of the current findings to other contexts.

3) Given ongoing discussions in the field how saliva oxytocin concentrations should be measured and interpreted, some more details on the current procedure should be added and discussed with respect to reported concerns in the literature.

4) The statistical analyses should be better justified and revised in accordance with the suggestions of Reviewer #1.

*Reviewer #1 (Recommendations for the authors):*

Schneider et al. investigated the association of affectionate touch and subjective well-being while also considering salivary cortisol and oxytocin as objective measures of stress responsiveness or buffering, respectively. The study was carried out during the first wave of the COVID-19 pandemic in Germany that was characterized by numerous restrictions of the social life to minimize the risk of infections. Following a cross-sectional online survey with more than 1,000 participants, nearly 250 subjects provided ecologically momentary assessment (EMA) data over two consecutive days including repeated measurements of salivary cortisol and oxytocin. Multilevel models revealed that the occurrence of affectionate touch was related to lower stress and increased happiness as well as lower cortisol levels across individuals. Moreover, momentary intensity of affectionate touch within individuals correlated negatively with general burden, stress, and anxiety and positively with happiness and oxytocin levels. There was no evidence for oxytocin mediating the effects of affectionate touch on objective (cortisol levels) or subjective stress responses (self-report ratings).

The current article has several strengths but also some weaknesses that are outlined below.

Strengths:

1. The current study including detailed EMA data as well as neuroendocrine variables nicely extends previous cross-sectional examinations that solely relied on self-report measures. This approach using large sample sizes allows for a comprehensive analysis of how affectionate touch is related to subjective well-being as well as salivary cortisol and oxytocin levels. Thereby, it seems possible not only to identify correlations between psychological constructs but also to address potential effects on a biological level.

2. The analyses are mostly adequate (see comments below) and consider a large amount of potential confounds.

3. The hypotheses, methods, and results are presented clearly and comprehensively, and I enjoyed reading the article.

Weaknesses:

1. Due to the correlational approach of the current study, explicit conclusions about the causality of effects are problematic. While it seems plausible that "affectionate touch might act as a buffer against anxiety and stress and might downregulate HPA axis outcomes" (line 305), there are also numerous alternative explanations for such relationship. For example, low levels of stress (e.g., caused by some unknown variable) might enhance social closeness and thereby modulate the occurrence and experience of affectionate touch as well as other psychological factors (e.g., anxiety or happiness). Alternatively, the mere presence of a loved one could be responsible for all the observed effects while at the same time also increasing the likelihood of affectionate touch. To consider such explanations, the discussion should be toned down.

2. The current study was conducted in a very specific context – the first wave of a pandemic that was associated with significant societal restrictions and uncertainties. While I fully agree that the current study is very relevant to understanding how affectionate touch might mitigate the deleterious mental health effects of such a situation, I was wondering to what extent the current findings could be generalized to other contexts. I would like to see at least a brief discussion of this issue.

3. While I appreciate that the authors carefully controlled for numerous potential confounds, I did not understand all relevant analysis steps. For example, why are random intercept and random intercept + random slopes models presented for all analyses? To my opinion, it would make more sense to only report the model that fits the data better (taking model complexity into account). Relatedly, the calculation of the time-rise (change from time point 1 to 3) and time-fall covariates (change from time point 3 to 6) seemed rather arbitrary to me. Figure 2 seems to indicate that time point 2 seems more appropriate than time point 3.

– In the abstract, it should be clarified whether the results describe between-person or within-person effects.

– In the section on "sample characteristics", it is mentioned that a rather large proportion of the sample indicated to suffer from a diagnosed mental disorder (>20%). I was wondering how representative such sample is?

– Table 1: The n's of the online survey participants (< 300) seem very low given that 1,050 participants provided data.

– Line 186: Covid-19 burden also failed to show a significant effect.

– Figure 3: Please use consistent terminology – "general burden" instead of "self-reported burden"

– Line 231: "general burden" instead of only "burden".

– Table 4 and 5: No model is marked with an asterisk, but the meaning of this symbol is still mentioned in the notes.

– Lines 312-315: Please double-check the results description. Significant effects were only obtained for general but not for Covid-19 related burden.

– Line 375: Typo "to report n conflicts of interest"?

– Line 391: What is meant by the sentence "To reduce potential noise in the data sampling was constantly monitored by study members."

– Line 432: The version of R is more relevant than the version of R Studio but I suppose that 4.1.1 already refers to the version of R.

*Reviewer #2 (Recommendations for the authors):*

The authors were seeking to investigate whether affectionate touch was associated with mental health during the pandemic using self-report measures and hormone levels.

A major strength of the paper was the large sample size. A weakness is that the study is observational so causation cannot be inferred. The authors achieved their aims and results support their conclusions. The paper is important as it shows the association between affectionate touch and lower distress during social isolation.

*Reviewer #3 (Recommendations for the authors):*

The research described in this paper mainly used online surveys which attempted to establish whether attitudes to and receipt of affectionate touch from others might act as a buffer to reduce stress in challenging circumstances with reduced social contact such as during the COVID-19 lockdowns.

The main strength of the study is in its large scale and systematic and rigorous analysis of the measures collected online and also the detailed analysis of behavioral and endocrine measures in a smaller cohort of subjects at multiple time points over two days. The main weakness resides in insufficient measures being included to clearly establish the importance of different types of affectionate touch, and their context, and whether other social (i.e. social interactions not involving touch) or physical factors (such as activity/exercise) likely to be influenced during the lockdown period showed similar associations with stress, anxiety and endocrine measures.

Overall, the findings reported by the authors suggest firstly that individuals with a positive attitude to social touch are more likely to exhibit stress and loneliness problems in circumstances where social contact is reduced, such as during the COVID-19 lockdowns. Secondly, findings provide some initial support for the potential importance of receiving affectionate touch in helping to reduce stress and anxiety and influence concentrations of hormones associated with their regulation (i.e. cortisol and oxytocin). However, further more detailed and controlled experiments will be needed to establish fully the specificity of these potentially beneficial effects of affectionate touch, the most important aspects and contexts of affectionate touch which are potentially influencing wellbeing and the mechanisms involved.

Touch, particularly in the form of massage, has well established anxiolytic and other effects on well-being, although the current study has extended this to demonstrate that the amount and intensity of interpersonal affectionate received in everyday contexts may also be important. Giving and receiving warm hugs and cuddles may be more important to our general mental wellbeing than we realize.

In terms of the measures recorded while the focus is on affectionate touch do the authors also have any data on other social (face to face conversations and other mutual activities not involving intimate touch) and physical activity likely to have some effect on cortisol and oxytocin concentrations?

While online information collection clearly has limitations concerning the amount of different variables which can be included I feel that given the central importance of the question concerning the role of receipt of affective touch many relevant details appear not to have been either included or considered. For example, were all subjects in a relationship and co-habiting/sleeping together? Was only one individual per household recruited? Given that a lot of affective touch can also be received from children and even pets, was this also considered? If subjects included a range of individuals both living alone and cohabiting I would have anticipated this being included as a variable, especially given the focus on COVID where individuals living alone might be expected to experience the greatest reduction in affective touch relative to those co-habiting/living in a family environment. As far as I can see there is no indication as to whether the subjects included in the study considered that they did receive less affectionate touch during the sampling period relative to before it?

The peak of oxytocin concentrations and reduced cortisol ones specifically at awakening is interesting but I wonder whether this simply reflects couples experiencing morning cuddles in a warm bed etc or a specific effect of awakening?

The reported saliva oxytocin concentrations are high and this is an ongoing issue in the field. I assume that the samples were run unextracted and also not in duplicate? While there is a clear recommendation now from both researchers and assay manufacturers to use extraction for plasma samples there is less consensus concerning saliva ones. With the widely used ELISA ENZO assay used in the current paper findings clearly shown that in extracted saliva samples generally result in baselines of <10pg/ml (see for example Fujii et al. 2016 Sci Reps 6:38662; Le et al. 2022 Psychother and Psychosom 91(5):335-347), while for unextracted ones this figure is much higher (up to 200 pg/ml), as in the current study. With plasma samples correlations between extracted and unextracted samples are weak, however a recent study comparing baseline and stimulated concentrations in saliva concentrations in post-partum women did report a significant low correlation (0.32-0.38) although profiles of release were different and concentrations were 10-fold higher in unextracted samples (Nagahasi-Araki et al. 2022, BMC Pregnancy and Childbirth 22:711). It is likely that higher concentrations in unextracted samples are contributed to by both the assay type (RIA vs ELISA) and whether the antibody used is sensitive to oxytocin fragments as well as oxytocin itself. Using a gold standard RIA, for example, as opposed to an ELISA, papers have consistently reported similar low oxytocin concentrations in both extracted plasma samples and unextracted saliva samples (generally below 5pg/ml – de Jong et al. 2015 Psychoneuroendocrinology 62:381-388; Martins et al., 2020 *eLife* 9:e62456), whereas with the ENZO ELISA extracted plasma and extracted saliva samples yield similar concentrations (Le et al., 2022). Indeed, there is a clear expectation that oxytocin concentrations should be similar in plasma and saliva since there is not reason to expect radically higher concentrations in saliva. The authors really need to at least acknowledge this issue and include it as a potential limitation. A recent review has, for example, highlighted the need for papers to make it very clear whether extracted or unextracted values are being reported (Tabak et al. 2023. Mol Psychiatry 28:127-140).

The authors do not mention a relevant study on humans reporting that foot massage both increases plasma oxytocin concentrations and corresponding neural responses in the orbitofrontal cortex and superior temporal sulcus but not somatosensory cortex. The study also reports that greater basal and manual touch-evoked oxytocin concentrations were associated with more positive attitudes to social touch (also using the STQ).(Li et al. 2019 Psychoneuroendocrinology 101:193-203).

While the authors have mainly decided to focus on presenting their findings and avoided entering into mechanistic explanations of how increased saliva oxytocin concentrations might influence levels of stress and anxiety etc I think it would help if there was a little more explanation of how saliva changes might influence the brain and endocrine system. In particular, the authors may wish to discuss the conclusion in another *eLife* paper that salivary and plasma oxytocin concentrations are not reliable trait markers for the oxytocin system in humans (Martins et al. 2020, *eLife* 9:62456).

In the discussion the authors state that affectionate touch reduced the COVID-19 related burden (line 313) but as far as I can see there was no significant effect found for this?

A small point but in the discussion of the study on rats by Tang et al. on line 326 animals are described as receiving "affectionate touch" from each other whereas perhaps simply receiving "social touch" might be more appropriate in this case.

# Reply: Author response

**DOI:** 10.7554/eLife.81241.sa2

Essential revisions:

1) The interpretation and conclusions of this study should be toned down as this purely correlative approach does not allow for drawing causal inferences about the relationship between affectionate touch, anxiety, stress, and hormone levels.

We thank the editor for this comment, and we agree that our study design does not allow causal conclusions. We, therefore, toned down the interpretation of our results and provided alternative possible explanations for the correlational results we find (p. 20-22).

2) It should be acknowledged that different types of affectionate touch and contexts might differentially affect wellbeing and hormone levels. Furthermore, other social and physical factors might have been affected by the restrictions during the first wave of the COVID-19 pandemic in Germany. Such potential confounds should be discussed in more detail with respect to the generalizability of the current findings to other contexts.

We agree with the editor and now more precisely discuss the potential effects of different kinds of touch on health-related outcomes and the generalizability of our results to non-pandemic contexts (p. 18- 22)

3) Given ongoing discussions in the field how saliva oxytocin concentrations should be measured and interpreted, some more details on the current procedure should be added and discussed with respect to reported concerns in the literature.

Measuring oxytocin concentrations in saliva and blood samples is indeed a highly discussed topic. We now address this topic in our manuscript, taking into account the most recently published review articles and discussing the descriptive rather than mechanistic findings we report here (p. 19-20)

4) The statistical analyses should be better justified and revised in accordance with the suggestions of Reviewer #1.

As suggested, we have thoroughly revised and adjusted the statistical approach where necessary (p. 9).

Reviewer #1 (Recommendations for the authors):

Schneider et al. investigated the association of affectionate touch and subjective well-being while also considering salivary cortisol and oxytocin as objective measures of stress responsiveness or buffering, respectively. The study was carried out during the first wave of the COVID-19 pandemic in Germany that was characterized by numerous restrictions of the social life to minimize the risk of infections. Following a cross-sectional online survey with more than 1,000 participants, nearly 250 subjects provided ecologically momentary assessment (EMA) data over two consecutive days including repeated measurements of salivary cortisol and oxytocin. Multilevel models revealed that the occurrence of affectionate touch was related to lower stress and increased happiness as well as lower cortisol levels across individuals. Moreover, momentary intensity of affectionate touch within individuals correlated negatively with general burden, stress, and anxiety and positively with happiness and oxytocin levels. There was no evidence for oxytocin mediating the effects of affectionate touch on objective (cortisol levels) or subjective stress responses (self-report ratings).

The current article has several strengths but also some weaknesses that are outlined below.

Strengths:

1. The current study including detailed EMA data as well as neuroendocrine variables nicely extends previous cross-sectional examinations that solely relied on self-report measures. This approach using large sample sizes allows for a comprehensive analysis of how affectionate touch is related to subjective well-being as well as salivary cortisol and oxytocin levels. Thereby, it seems possible not only to identify correlations between psychological constructs but also to address potential effects on a biological level.

2. The analyses are mostly adequate (see comments below) and consider a large amount of potential confounds.

3. The hypotheses, methods, and results are presented clearly and comprehensively, and I enjoyed reading the article.

Weaknesses:

1. Due to the correlational approach of the current study, explicit conclusions about the causality of effects are problematic. While it seems plausible that "affectionate touch might act as a buffer against anxiety and stress and might downregulate HPA axis outcomes" (line 305), there are also numerous alternative explanations for such relationship. For example, low levels of stress (e.g., caused by some unknown variable) might enhance social closeness and thereby modulate the occurrence and experience of affectionate touch as well as other psychological factors (e.g., anxiety or happiness). Alternatively, the mere presence of a loved one could be responsible for all the observed effects while at the same time also increasing the likelihood of affectionate touch. To consider such explanations, the discussion should be toned down.

We want to thank the reviewer for this constructive comment. We agree that with this nonexperimental design causal interpretations are critical, and the data must be interpreted cautiously. Since in this data assessment in everyday life, affectionate touch could not be experimentally manipulated and the results are correlational, we agree that causal conclusions are difficult to draw. We have therefore rephrased our conclusions (p. 18):

“Note that in this data assessment in everyday life, affectionate touch could not be experimentally manipulated and these correlative results should be interpreted with caution…”

As suggested, we have included alternative explanations in the Discussion section (p. 21):

” Alternatively, it is also possible that initially higher levels of participants’ well-being (including lower stress, anxiety, or burden) might have increased feelings of closeness and therefore promoted affectionate touch. Also, other aspects that accompany physical contact, such as eye contact, compliments, or affective closeness with loved ones could have contributed to the beneficial effects.”

2. The current study was conducted in a very specific context – the first wave of a pandemic that was associated with significant societal restrictions and uncertainties. While I fully agree that the current study is very relevant to understanding how affectionate touch might mitigate the deleterious mental health effects of such a situation, I was wondering to what extent the current findings could be generalized to other contexts. I would like to see at least a brief discussion of this issue.

We thank the reviewer for bringing up this very important aspect of our data. On the one hand, the pandemic lockdown strengthens the impact of our results due to the clinically relevant and ecologically valid stress situation. On the other hand, it is (luckily and hopefully) a very uncommon and rare occurrence, which clearly raises the question of how to interpret the data in light of other situations. We now address this question in our manuscript (p. 22):

“While the situation was quite specific and limited the generalizability of the results to everyday life in pre- or post-pandemic conditions, the risk of viral infection was not the only concern among the population. Participants reported significant concerns about being isolated from others and how long it might take for them to get back to normal (Hopf et al., 2021). These concerns are related, at least in part, to the fear of loneliness, defined as a perceived lack of social connection and the distress this causes (Bekhet et al., 2008). Thus, these results obtained in the general population during pandemic-related restrictions can be partially transferred to other situations, such as a lack of social contacts due to migration, physical illnesses/disabilities, or other reasons.”

3. While I appreciate that the authors carefully controlled for numerous potential confounds, I did not understand all relevant analysis steps. For example, why are random intercept and random intercept + random slopes models presented for all analyses? To my opinion, it would make more sense to only report the model that fits the data better (taking model complexity into account). Relatedly, the calculation of the time-rise (change from time point 1 to 3) and time-fall covariates (change from time point 3 to 6) seemed rather arbitrary to me. Figure 2 seems to indicate that time point 2 seems more appropriate than time point 3.

We appreciate this reviewer’s comment. For the sake of transparency, we initially reported all random intercept and fixed slopes as well as random intercepts and random slopes models in the previous version of this manuscript. However, we agree with the reviewer that it makes sense to optimize and reduce the number of reported models. To keep the report consistent, we decided to report random intercept and random slopes models on affectionate touch (yes/no), since four of the seven models showed a statistically better fit when the slopes were set at random. For models with the intensity of touch as the outcome variable, we now report random intercepts and fixed slopes models (none of these models showed a better model fit when slopes were set at random). The following information is now in the statistical analyses section (p. 9):

“For the models on affectionate touch as a binary variable (yes/no), we report random intercept and random slopes models in the Results section, since these showed a statistically better fit as compared to random intercept and fixed slopes models. However, for the dimensional intensity of affectionate touch, we report random intercept and fixed slopes models, since the random slope models did not yield a better model fit.”

Regarding the time-rise (change from time point 1 to 3) and time-fall (change from time point 3 to 6), we initially made this decision based on the theoretical assumption that the first three time points (all scheduled within 45 minutes after awakening) represent the typical cortisol awakening response (CAR), which represents a distinct process from the diurnal change throughout the rest of the day (we relied on tutorials on how to calculate and interpret CAR (Stalder et al., 2016)). We had therefore made the a-priori decision for a separate representation of the cortisol awakening response in our statistical models. However, after re-evaluating our decision in response to this comment, we realized that the first two time points are irrelevant to our research question because affectionate touch was not assessed at the first two time points of a day (only at time points 3-6). Consequently, there is no need to control for the initial rise in cortisol from time point 1 to 3 in our models. We have therefore adjusted our models and removed the covariate representing time-rise from our multilevel models. These models now contain only a time variable representing the linear decrease in cortisol levels throughout the day after the cortisol awakening response.

– In the abstract, it should be clarified whether the results describe between-person or within-person effects.

We thank the reviewer for this comment and have included information on within-person and between-person effects in the abstract.

– In the section on "sample characteristics", it is mentioned that a rather large proportion of the sample indicated to suffer from a diagnosed mental disorder (>20%). I was wondering how representative such sample is?

We agree that these are high numbers of self-reported mental impairment, indeed. However, the pre-pandemic 12-month prevalence of any psychiatric disease has been estimated to be about 28% (Jacobi et al., 2014). Thus, the observed prevalence rate of about 20% is, actually, even lower than the rate reported for the general population in Germany. As we believe that this is an important point, we now comment on this data in the Discussion section (p.17):

"However, it is further important to note that about 20% of the participants reported having at least one psychiatric diagnosis. In comparison, the pre-pandemic 12-month prevalence in the general population in Germany is about 28 % (Jacobi et al., 2014). Thus, our sample seems to be slightly less burdened compared to the general population, which partly limits the generalizability of the results."

– Table 1: The n's of the online survey participants (< 300) seem very low given that 1,050 participants provided data.

The reviewer might have misunderstood the first column as the total sample: n = 227, which refers to the number of male participants, whereas n = 815 refers to the number of female participants. We now have changed the presentation of the numbers in table 1.

– Line 186: Covid-19 burden also failed to show a significant effect.

We apologize for this mistake and changed it in the text.

– Figure 3: Please use consistent terminology – "general burden" instead of "self-reported burden"

Thanks for pointing out the inconsistent wording, we have changed it in the figure.

– Line 231: "general burden" instead of only "burden".

Done.

– Table 4 and 5: No model is marked with an asterisk, but the meaning of this symbol is still mentioned in the notes.

We have removed the asterisk from the table notes.

– Lines 312-315: Please double-check the results description. Significant effects were only obtained for general but not for Covid-19 related burden.

Thanks for pointing this out, we have corrected it in the text.

– Line 375: Typo "to report n conflicts of interest"?

We corrected the typo.

– Line 391: What is meant by the sentence "To reduce potential noise in the data sampling was constantly monitored by study members."

We changed the wording and made additions to the monitoring description to make it clearer: “To reduce potential missing values, minimize irregularities and to increase adherence, the data sampling was constantly monitored by study members.”

– Line 432: The version of R is more relevant than the version of R Studio but I suppose that 4.1.1 already refers to the version of R.

We agree with the reviewer’s comment on R, we, in fact, used R version 4.1.1 and changed it within the text: “Statistical analyses were conducted using R studio (R version 4.1.1).”

Reviewer #2 (Recommendations for the authors):

The authors were seeking to investigate whether affectionate touch was associated with mental health during the pandemic using self-report measures and hormone levels.

A major strength of the paper was the large sample size. A weakness is that the study is observational so causation cannot be inferred. The authors achieved their aims and results support their conclusions. The paper is important as it shows the association between affectionate touch and lower distress during social isolation.

We thank the reviewer for this positive feedback!

Reviewer #3 (Recommendations for the authors):

The research described in this paper mainly used online surveys which attempted to establish whether attitudes to and receipt of affectionate touch from others might act as a buffer to reduce stress in challenging circumstances with reduced social contact such as during the COVID-19 lockdowns.

The main strength of the study is in its large scale and systematic and rigorous analysis of the measures collected online and also the detailed analysis of behavioral and endocrine measures in a smaller cohort of subjects at multiple time points over two days. The main weakness resides in insufficient measures being included to clearly establish the importance of different types of affectionate touch, and their context, and whether other social (i.e. social interactions not involving touch) or physical factors (such as activity/exercise) likely to be influenced during the lockdown period showed similar associations with stress, anxiety and endocrine measures.

Overall, the findings reported by the authors suggest firstly that individuals with a positive attitude to social touch are more likely to exhibit stress and loneliness problems in circumstances where social contact is reduced, such as during the COVID-19 lockdowns. Secondly, findings provide some initial support for the potential importance of receiving affectionate touch in helping to reduce stress and anxiety and influence concentrations of hormones associated with their regulation (i.e. cortisol and oxytocin). However, further more detailed and controlled experiments will be needed to establish fully the specificity of these potentially beneficial effects of affectionate touch, the most important aspects and contexts of affectionate touch which are potentially influencing wellbeing and the mechanisms involved.

Touch, particularly in the form of massage, has well established anxiolytic and other effects on well-being, although the current study has extended this to demonstrate that the amount and intensity of interpersonal affectionate received in everyday contexts may also be important. Giving and receiving warm hugs and cuddles may be more important to our general mental wellbeing than we realize.

We appreciate the reviewer’s thorough assessment of our work and the constructive comments.

In terms of the measures recorded while the focus is on affectionate touch do the authors also have any data on other social (face to face conversations and other mutual activities not involving intimate touch) and physical activity likely to have some effect on cortisol and oxytocin concentrations?

We thank the reviewer for this question. We included the variable of physical activity in our initial models to control for its potential influence on hormonal outcomes and did not find any significant associations. Indeed, in this paper we focused on the associations of affectionate touch in times of physical contact restrictions. To avoid α error inflation, originally, we did not conduct exploratory analyses. However, in response to the reviewer’s comment, we ran additional analyses on a specific other social activity. We used conversations as a predictor, as we think this could serve as an interesting comparison to touch. We analyzed whether a conversation (yes/no) was associated with hormonal levels and did not find significant associations, neither with cortisol nor oxytocin levels. In the following and equivalent to the touch data, we analyzed the associations of hormonal levels with the intensity of conversations and found a significant positive link between the intensity of conversation and oxytocin levels on within-person level, but not with cortisol concentrations. However, it must be noted that both variables, conversations, and affectionate touch, are highly correlated. If the editor and reviewer recommend us to report these findings, we can add the analyses in supplementary files to the manuscript.

While online information collection clearly has limitations concerning the amount of different variables which can be included I feel that given the central importance of the question concerning the role of receipt of affective touch many relevant details appear not to have been either included or considered. For example, were all subjects in a relationship and co-habiting/sleeping together? Was only one individual per household recruited? Given that a lot of affective touch can also be received from children and even pets, was this also considered? If subjects included a range of individuals both living alone and cohabiting I would have anticipated this being included as a variable, especially given the focus on COVID where individuals living alone might be expected to experience the greatest reduction in affective touch relative to those co-habiting/living in a family environment. As far as I can see there is no indication as to whether the subjects included in the study considered that they did receive less affectionate touch during the sampling period relative to before it?

Although we asked participants whether they received affectionate touch including hugs, kisses, cuddles, caresses, etc., it is not possible to extract the source of touch from this data.

Regarding relationship and cohabitation status, about 29 % of the subjects were single and 21% were living alone. We did not get into more detail within this paper as the focus of this study was to analyze within-subject associations of affectionate touch and hormonal and self-report measures. Nevertheless, we agree with the reviewer that living arrangements and relationship status might make a difference, especially during Covid-19 lockdowns, where social touch outside the own household was not permitted. In response to the reviewer’s comment, we considered the variables in our analyses. The group of individuals living with others reported more affectionate touch (514 times) than individuals living alone (42 times). Similarly, those in a relationship reported more affectionate touch (525 times) than singles (30 times). Within-subject association between touch, cortisol or oxytocin did not change overall, when relationship status or living situation were included. However, the between-subject association between affectionate touch and cortisol was mainly driven by the group with romantic partner. These results are linked with the reviewer’s earlier comment and suggests that those in a romantic relationship exchanged other types and more intense and erotic touch than singles – which again is in line with the association of touch intensity and oxytocin found here.

We unfortunately have only indirect information on whether exchanged touch had changed with the pandemic: via the online assessment participants were asked whether the quality of social contacts had changed since the beginning of the pandemic. Within the EMA sample, about 71% of the participants reported that the perceived quality of their social contacts was lower compared to before the pandemic. Based on this item, we cannot conclude whether the affectionate touch was received less often, we can only speculate that the reported reduction in the quality of social contact could possibly represent the reduction of affectionate touch.

The peak of oxytocin concentrations and reduced cortisol ones specifically at awakening is interesting but I wonder whether this simply reflects couples experiencing morning cuddles in a warm bed etc or a specific effect of awakening?

We thank the reviewer for bringing up this important question. To test this hypothesis, we now graphed oxytocin levels throughout the day, but separated the data based on the participants’ living situation relationship status (single vs. in a relationship) or (living alone vs. living with others). Of note, the patterns look almost identical (Figure 2—figure supplement 1). Even though we cannot rule out that there might be some other (unknown) factors responsible for the oxytocin “awakening response”, it seems that it is not exclusively due to the presence of a loved one.

We now also mention this in the manuscript (p. 13) and add these graphs as a supplement to our manuscript:

“The patterns of daily profiles did not appear to differ based on participants’ relationship status (single vs. in a relationship) or living situation (alone vs. with others).”

The reported saliva oxytocin concentrations are high and this is an ongoing issue in the field. I assume that the samples were run unextracted and also not in duplicate? While there is a clear recommendation now from both researchers and assay manufacturers to use extraction for plasma samples there is less consensus concerning saliva ones. With the widely used ELISA ENZO assay used in the current paper findings clearly shown that in extracted saliva samples generally result in baselines of <10pg/ml (see for example Fujii et al. 2016 Sci Reps 6:38662; Le et al. 2022 Psychother and Psychosom 91(5):335-347), while for unextracted ones this figure is much higher (up to 200 pg/ml), as in the current study. With plasma samples correlations between extracted and unextracted samples are weak, however a recent study comparing baseline and stimulated concentrations in saliva concentrations in post-partum women did report a significant low correlation (0.32-0.38) although profiles of release were different and concentrations were 10-fold higher in unextracted samples (Nagahasi-Araki et al. 2022, BMC Pregnancy and Childbirth 22:711). It is likely that higher concentrations in unextracted samples are contributed to by both the assay type (RIA vs ELISA) and whether the antibody used is sensitive to oxytocin fragments as well as oxytocin itself. Using a gold standard RIA, for example, as opposed to an ELISA, papers have consistently reported similar low oxytocin concentrations in both extracted plasma samples and unextracted saliva samples (generally below 5pg/ml – de Jong et al. 2015 Psychoneuroendocrinology 62:381-388; Martins et al., 2020 eLife 9:e62456), whereas with the ENZO ELISA extracted plasma and extracted saliva samples yield similar concentrations (Le et al., 2022). Indeed, there is a clear expectation that oxytocin concentrations should be similar in plasma and saliva since there is not reason to expect radically higher concentrations in saliva. The authors really need to at least acknowledge this issue and include it as a potential limitation. A recent review has, for example, highlighted the need for papers to make it very clear whether extracted or unextracted values are being reported (Tabak et al. 2023. Mol Psychiatry 28:127-140).

We thank the reviewer for these thoughtful comments. We agree that the information about whether the saliva samples were extracted prior to analyses is important and included this information in the methods section. We further discuss the methodological issues and debates regarding peripheral measures of oxytocin and include the recent reviews of Tabak et al., 2023 and Martins et al., 2020 that were mentioned by the reviewer (p. 19-20):

“Moreover, in the last decade, there has been an extensive discussion about the reliability and validity of peripheral oxytocin measures (Martins et al., 2020, Szeto et al., 2011, Tabak et al., 2023). There are several methodological issues and challenges associated with measuring oxytocin in blood plasma and saliva samples (Tabak et al., 2023). For example, studies have shown that oxytocin concentrations after sample extraction are much lower compared to unextracted oxytocin measurements (Szeto et al., 2011). Additionally, the correlations between extracted and unextracted oxytocin levels as well as between saliva and plasma oxytocin concentrations have been inconsistent across studies (e.g. Hoffman et al., 2012, Martins et al., 2020, Nagahashi-Araki et al., 2022, Szeto et al., 2011). These inconsistencies might be due to numerous reasons including differences in the study population, methods of sample processing and analyses. In particular, using different assay types (e.g. radioimmunoassay vs. enzyme immunoassay), as well as sample preparation (extraction vs. non-extraction), may contribute to these inconsistencies and make it difficult to compare results between studies (Tabak et al., 2023). Since unextracted samples were used in this study, the concentrations probably represent both free and bound oxytocin (MacLean et al., 2019) thereby potentially limiting the comparability with studies using extracted samples. Another important issue is the intraindividual stability of oxytocin over time (Feldman et al., 2013, Martins et al., 2020, Schneiderman et al., 2012). A recent study reports no correlation of single oxytocin measures between several assessments indicating that single measures of oxytocin might be not reliable to represent oxytocin baseline levels (Martins et al., 2020). In our sample, we found a significant positive correlation of mean oxytocin values between the two assessment days. As fluctuations of oxytocin throughout the day were apparent in our study, correlating mean values of six oxytocin measures over the day might represent the individual baseline oxytocin levels better than single measures (this has also been discussed in Tabak et al., 2023). However, the difference between our data and the findings reported by Martins et al., 2020 might also reflect methodological differences, as previous studies with unextracted samples also reported positive correlations over time (Feldman et al., 2013, Schneiderman et al., 2012).”

The authors do not mention a relevant study on humans reporting that foot massage both increases plasma oxytocin concentrations and corresponding neural responses in the orbitofrontal cortex and superior temporal sulcus but not somatosensory cortex. The study also reports that greater basal and manual touch-evoked oxytocin concentrations were associated with more positive attitudes to social touch (also using the STQ).(Li et al. 2019 Psychoneuroendocrinology 101:193-203).

We thank the reviewer for bringing this relevant study to our attention and have now included this paper in our introduction (p. 4):

“Similarly, a more recent study found a significant increase in plasma oxytocin and corresponding neural responses after a foot massage. Interestingly, basal oxytocin concentrations, as well as oxytocin increase after the massage, were associated with more positive attitudes towards social touch.”

As well as in the Discussion section (p. 18):

“Interestingly, a recent study demonstrated that foot massage was rated as more pleasurable and rewarding and was associated with a higher increase of oxytocin after the massage administered by hand, as compared to machine-administered massage, although the intensity of the massage was rated similarly (Li et al., 2019).”

While the authors have mainly decided to focus on presenting their findings and avoided entering into mechanistic explanations of how increased saliva oxytocin concentrations might influence levels of stress and anxiety etc I think it would help if there was a little more explanation of how saliva changes might influence the brain and endocrine system. In particular, the authors may wish to discuss the conclusion in another eLife paper that salivary and plasma oxytocin concentrations are not reliable trait markers for the oxytocin system in humans (Martins et al. 2020, eLife 9:62456).

We agree with the reviewer that this is an additional important point of debate. We are thankful to you for bringing up this paper. One of the arguments, that oxytocin measures in the periphery are not reliable, was that the authors did not find any correlation between oxytocin levels from different time points. We tested how the mean values from day 1 were correlated with day 2 and found a high correlation for both oxytocin and cortisol between days. We have now included that information in the manuscript (p. 13):

“A positive correlation between the two assessment days was found for individual (ln-transformed) mean values of oxytocin (r(227)=.850, p<.001) as well as cortisol (r(243)=.571, p<.001) levels.” We further discuss this paper, along with other papers regarding methodological issues of oxytocin assessments, in our Discussion section (text in the comment above).

We think that our data suggest a specific diurnal pattern in salivary oxytocin, which might have not been captured in previous studies. However, please note that we interpret salivary oxytocin levels as a consequence, rather than a driving factor of central nervous system phenomena, such as anxiety, stress or HPA axis activation. Recently, there has been data on touch and central nervous activation of oxytocin in female rats (Tang et al., 2020). We assume that such dynamics might predict peripheral oxytocin increases. We discuss this in more detail in the Discussion section (p. 21).

In the discussion the authors state that affectionate touch reduced the COVID-19 related burden (line 313) but as far as I can see there was no significant effect found for this?

We agree with the reviewer and corrected this mistake by changing the word “Covid-19 related” into “general”.

A small point but in the discussion of the study on rats by Tang et al. on line 326 animals are described as receiving "affectionate touch" from each other whereas perhaps simply receiving "social touch" might be more appropriate in this case.

We agree that in this case “social touch” is more appropriate and exchanged the words in the manuscript.

Additionally added references:

Bekhet, A. K., Zauszniewski, J. A., & Nakhla, W. E. (2008). Loneliness: a concept analysis. Nursing forum,

Feldman, R., Gordon, I., Influs, M., Gutbir, T., & Ebstein, R. P. (2013). Parental oxytocin and early caregiving jointly shape children’s oxytocin response and social reciprocity. Neuropsychopharmacology, 38(7), 1154-1162.

Hoffman, E. R., Brownley, K. A., Hamer, R. M., & Bulik, C. M. (2012). Plasma, salivary, and urinary oxytocin in anorexia nervosa: a pilot study. Eating Behaviors, 13(3), 256-259.

Hopf, D., Schneider, E., Eckstein, M., Aguilar-Raab, C., & Ditzen, B. (2021). COVID-und Social Distancing bezogene Sorgen und ihre Beziehung zu psychischer und körperlicher Erkrankung. PPmPPsychotherapie· Psychosomatik· Medizinische Psychologie, 71(02), 57-60.

Jacobi, F., Höfler, M., Strehle, J., Mack, S., Gerschler, A., Scholl, L., Busch, M. A., Maske, U., Hapke, U., & Gaebel, W. (2014). Psychische störungen in der allgemeinbevölkerung. Nervenarzt, 85(1), 77-87.

Li, Q., Becker, B., Wernicke, J., Chen, Y., Zhang, Y., Li, R., Le, J., Kou, J., Zhao, W., & Kendrick, K. M. (2019). Foot massage evokes oxytocin release and activation of orbitofrontal cortex and superior temporal sulcus. Psychoneuroendocrinology, 101, 193-203.

MacLean, E. L., Wilson, S. R., Martin, W. L., Davis, J. M., Nazarloo, H. P., & Carter, C. S. (2019). Challenges for measuring oxytocin: The blind men and the elephant? Psychoneuroendocrinology, 107, 225-231.

Martins, D., Gabay, A. S., Mehta, M., & Paloyelis, Y. (2020). Salivary and plasmatic oxytocin are not reliable trait markers of the physiology of the oxytocin system in humans. *ELife*, 9, 1-19. https://doi.org/https://doi.org/10.7554/*eLife*.62456

Musardo, S., Contestabile, A., Knoop, M., Baud, O., & Bellone, C. (2022). Oxytocin neurons mediate the effect of social isolation via the VTA circuits. *ELife*, 11.

Nagahashi-Araki, M., Tasaka, M., Takamura, T., Eto, H., Sasaki, N., Fujita, W., Miyazaki, A., Morifuji, K., Honda, N., & Miyamura, T. (2022). Endogenous oxytocin levels in extracted saliva elevates during breastfeeding correlated with lower postpartum anxiety in primiparous mothers. BMC Pregnancy and Childbirth, 22(1), 1-10.

Quintana, D. S., Rokicki, J., van der Meer, D., Alnæs, D., Kaufmann, T., Córdova-Palomera, A., Dieset, I., Andreassen, O. A., & Westlye, L. T. (2019). Oxytocin pathway gene networks in the human brain. Nature communications, 10(1), 1-12.

Schneiderman, I., Zagoory-Sharon, O., Leckman, J. F., & Feldman, R. (2012). Oxytocin during the initial stages of romantic attachment: relations to couples’ interactive reciprocity. Psychoneuroendocrinology, 37(8), 1277-1285.

Stalder, T., Kirschbaum, C., Kudielka, B. M., Adam, E. K., Pruessner, J. C., Wüst, S., Dockray, S., Smyth, N., Evans, P., & Hellhammer, D. H. (2016). Assessment of the cortisol awakening response: Expert consensus guidelines. Psychoneuroendocrinology, 63, 414-432.

Szeto, A., McCabe, P. M., Nation, D. A., Tabak, B. A., Rossetti, M. A., McCullough, M. E., Schneiderman, N., & Mendez, A. J. (2011). Evaluation of enzyme immunoassay and radioimmunoassay methods for the measurement of plasma oxytocin. Psychosomatic medicine, 73(5), 393-400.

Tabak, B. A., Leng, G., Szeto, A., Parker, K. J., Verbalis, J. G., Ziegler, T. E., Lee, M. R., Neumann, I. D., & Mendez, A. J. (2023). Advances in human oxytocin measurement: Challenges and proposed solutions. Molecular psychiatry, 28(1), 127-140.

Tang, Y., Benusiglio, D., Lefevre, A., Hilfiger, L., Althammer, F., Bludau, A., Hagiwara, D., Baudon, A., Darbon, P., Schimmer, J., Kirchner, M. K., Roy, R. K., Wang, S., Eliava, M., Wagner, S., Oberhuber, M., Conzelmann, K. K., Schwarz, M., Stern, J. E.,... Grinevich, V. (2020). Social touch promotes interfemale communication via activation of parvocellular oxytocin neurons. Nature neuroscience, 23(9), 1125-1137.
